# TNF-****α**** Polymorphisms in Juvenile Idiopathic Arthritis: Which Potential Clinical Implications?

**DOI:** 10.1155/2012/756291

**Published:** 2012-10-21

**Authors:** A. Scardapane, L. Breda, M. Lucantoni, F. Chiarelli

**Affiliations:** Department of Pediatrics, University of Chieti, Via Vestini 5, 66100 Chieti, Italy

## Abstract

Whether tumor necrosis factor alpha (TNF-**α**) gene polymorphisms (SNPs) influence disease susceptibility and treatment of patients with juvenile idiopathic arthritis (JIA) is presently uncertain. TNF-**α** is one of the most important cytokine involved in JIA pathogenesis. Several single nucleotide polymorphisms (SNPs) have been identified within the region of the TNF-**α** gene but only a very small minority have proven functional consequences and have been associated with susceptibility to JIA. An association between some TNF-**α** SNPs and adult rheumatoid arthritis (RA) susceptibility, severity and clinical response to anti-TNF-**α** treatment has been reported. The most frenquetly studied TNF-**α** SNP is located at −308 position, where a substitution of the G allele with the rare A allele has been found. The presence of the allele −308A is associated to JIA and to a poor prognosis. Besides, the −308G genotype has been associated with a better response to anti-TNF-**α** therapy in JIA patients, confirming adult data. Psoriatic and oligoarticular arthritis are significantly associated to the −238 SNP only in some works. Studies considering other SNPs are conflicting and inconclusive. Large scale studies are required to define the contribution of TNF-**α** gene products to disease pathogenesis and anti-TNF-**α** therapeutic efficacy in JIA.

## 1. Introduction

Juvenile idiopathic arthritis (JIA) is the most common chronic rheumatic disease of childhood [1]. It is an heterogeneous disease group of unknown aetiology with distinct presentation, clinical features, and genetic background [2]. JIA is a complex genetic disease caused by the effects of environmental factors and multiple genes that act in concert to predispose the host to the development of JIA and to determine the different disease phenotypes [3]. Common to all JIA subgroups is the chronic inflammation within synovial joints [4]. Cytokines, a group of modulatory proteins or glycoproteins produced by a wide range of cells in response to a variety of stimuli, are important mediators and regulators of synovial inflammation [5]. Basal and cell-stimulated cytokine levels differ between individuals; both genetic and environmental influences have been shown to play a role in their variability [6]. Genetic variation that produces altered structure or expression of a cytokine can have evident pathological consequences, as chronic diseases, increased risk of infection, and altered outcome of acute disorders [7]. Variations in DNA include single nucleotide polymorphisms (SNPs), microsatellites, and insertion/deletion polymorphisms. A large number of polymorphisms within the coding and noncoding regions of cytokines genes have been identified, and several thousand disease-association studies have been carried out using these variants [6]. Defining cytokine gene polymorphisms in JIA is linked to the necessity of understanding the aetiology, discovering possible markers of severity, and identifying targets for therapeutic intervention. Some cytokine genes are strongly involved in the pathogenesis of JIA [4]. One of the most important molecule is TNF-*α*, several studies have demonstrated high TNF-*α* levels in both sera and synovial fluid of children with chronic arthritis [8–10]. Several SNPs have been identified within the promoter, exonic, intronic, and 3′-untranslated region of the TNF-*α* gene, but conflicting results have been reported [11, 12]. Although many SNPs have been reported, only a very small minority of the genetic variants published have proven functional consequences and have been associated with susceptibility to JIA. Furthermore, associations between TNF-*α* polymorphisms and subtypes of JIA have been found [13–16]. Another important remark is pharmacogenetic applications of cytokine SNPs. In the last ten years, biologic drugs have been introduced in the treatment of JIA. Among these drugs TNF-*α* antagonists (etanercept, infliximab, and adalimumab) play a primary role [17]. Etanercept has proven highly efficacious in children with polyarticular JIA and is a Food and Drug Administration (FDA) approved drug for these patients [18].

Unfortunately, there is a group of patients, defined as nonresponders, that has no benefit from TNF-*α* blockade and has a worst prognosis. Defining the gene responsible for the phenotype might allow the identification of subjects who could benefit from a specific therapeutic intervention. 

At this regard, most medical literature data are based on studies performed in adult rheumatoid arthritis (RA) patients and few studies in JIA subjects [15, 20–23]. 

The aim of this study is to collect the present knowledge about the principal TNF-*α* gene polymorphisms in JIA, their possible role in the pathogenesis, the severity, and the response to drugs.

## 2. Methods

In this work we reviewed the current knowledge regarding the role of TNF-*α* polymorphisms in JIA with focus on the evidence for pathogenesis, phenotypes, prognosis, and therapeutic response to anti-TNF-*α* drugs.

### 2.1. Search Strategy

Using PubMed from the International Library of Medicine, relevant literature on the role of TNF-*α* polymorphisms in JIA from January 1990 to November 2010 was reviewed. Five researches were performed for the keywords: “tumor necrosis factor alpha polymorphism,” “juvenile arthritis and tumor necrosis factor alpha,” “juvenile arthritis and tumor necrosis factor alpha polymorphism,” “tumor necrosis factor alpha and arthritis therapy,” and “tumor necrosis factor alpha polymorphism and arthritis therapy.” Language was restricted to English. 

### 2.2. Selection of Studies

The first selection reviewed the titles and the abstracts for all the articles retrieved. The titles and abstracts not closely related to our research were excluded. Expert opinion and case-report works were not included. 

### 2.3. Data Collected

Using the first keyword, “tumor necrosis factor alpha polymorphism,” we found 2794 articles and selected 88 of these; for the second research we used the keyword “juvenile arthritis and tumor necrosis factor alpha” and found 327 works and of these 54 articles were considered. For the third keyword, “juvenile arthritis and tumor necrosis factor alpha polymorphism,” we detected 22 articles and considered 14 works. The fourth research “tumor necrosis factor alpha and arthritis therapy” detected 4864 and selected 81 of these. The last search “tumor necrosis factor alpha polymorphism and arthritis therapy” found 69 works and selected 33 of them. Finally, a further review of all selected articles led us to choose a total of 105 works.

## 3. Results

### 3.1. Juvenile Arthritis Pathogenesis

Juvenile idiopathic arthritis (JIA) is a group of chronic arthritides of unknown aetiology occurring in children under the age of 16 years [24]. In the United States, JIA has an estimated prevalence of 16–150 per 100,000 children [25]. The International League of Association for Rheumatology (ILAR) defines seven subtypes of JIA that exhibit differences in age at onset, clinical features, prognosis, and genetic background. The oligoarticular type is characterized by the involvement of one to four joints (monoarticular or oligoarticular); the polyarticular type involves five or more joints; the systemic arthritis is characterized by the presence of fever and systemic involvement at onset. The diagnosis of juvenile psoriatic arthritis need the presence of arthritis and a typical psoriatic rash or a family history of psoriasis. Another form encompasses the arthritides associated with enthesitis. In some patients arthritis may involve the sacroiliac and spinal joints thus producing the clinical picture of ankylosing spondylitis [2, 4]. All JIA subtypes are characterized by persistent joint swelling caused by accumulation of synovial fluid and thickening of the synovial lining [24]. The aetiology of JIA is still poorly understood; the heterogeneity of this disease implies that different factors probably contribute to the pathogenesis. Significantly elevation of sera and synovial levels of proinflammatory cytokines and the presence of autoantibodies in JIA subjects suggest the involvement of the immune system [26–28]. The results of genome-wide association studies (GWAS) in children and sibling recurrence risk in family studies support the assumption that multiple genes probably contribute to JIA susceptibility: it is accepted that an environmental factor (i.e., infections) triggers the disease in genetically predisposed subjects [3, 4, 24, 29].

First genetic investigations focused on the human leucocyte antigens (HLA) within the major histocompatibility complex (MHC) loci on the sixth chromosome. MHC genes are highly conserved sequences of genome that present a certain variability which cause an individual response to various stimuli. For this reason a variable mechanism of starting and maintenance of the inflammatory response exists in different individuals. Particular associations between specific HLA alleles and different JIA subtypes have been found. The strongest association has been observed in the oligoarthritis subgroup, in particular with the alleles HLA-DRB1*01, HLA-DRB1*11 (DR5), HLA-DRB1*08 (DR8), HLA-DRB1*13 (DR6), and HLA-DPB*02. Positive rheumatoid factor polyarthritis has been associated with HLA-DR4 and HLA-DRB1*11; negative rheumatoid factor polyarthritis has been demonstrated to be associated with HLA-DRB1*08 and HLA-DPB1*03 [30, 31]. The HLA-DRB1*04 has been linked to systemic arthritis [3, 32] and the HLAB27 have long been recognised as a contributing factor to the development of enthesitis related arthritis; moreover, this allele seems to be related to axial inflammation with hip involvement and subsequent juvenile ankylosing spondylitis [3, 4, 26, 33, 34]. Several other genes are probably involved in JIA pathogenesis; indeed elevated synovial and sera levels of TNF-*α* and other inflammatory cytokines as interleukin-1 (IL-1), interleukin-6 (IL-6), and interferon-gamma (INF-*γ*) in JIA subjects are likely related to abnormal expression of genes for cytokine production and regulation [26, 33].

Several studies have focused blood and synovial cytokine polymorphisms; a major involved cytokine in JIA is the TNF-*α*; the polymorphisms of its gene have been evaluated in different studies to establish its role in the pathogenesis and in the therapeutic response to anti-TNF-*α* drugs [4, 6, 7, 35, 36].

### 3.2. TNF-*α*

TNF-*α* is a proinflammatory cytokine produced as a membrane-bound 26 kDa molecule from which the soluble 17 kDa active TNF-*α* molecule is released by the TNF-*α* converting enzyme (TACE) [37]. The circulating TNF-*α* levels are highly variable [38]. 

TNF-*α* is involved in several biologic processes such as tissue remodelling, epithelial cell barrier permeability, macrophage activation, recruitment of inflammatory cells, effectiveness of the local and systemic inflammation, and amplification of other proinflammatory cytokine actions [39–41]. The biological functions of TNF-*α* have been demonstrated to be related to the concentration and the duration of exposure to TNF-*α* molecule. In the acute situation, local production of TNF-*α* have a clear positive action increasing the expression of adhesion molecules on the vascular endothelium to allow immune cells, as macrophages and neutrophils, to reach the sites of tissue damage or infection. Furthermore, TNF-*α* activates phagocytes to clear infectious agents and cellular debris [37, 41]. On the other hand, systemic or protracted exposure to TNF-*α* may be harmful. The upregulation of TNF-*α* gene expression has been involved in the pathogenesis of several autoimmune inflammatory illnesses, such as systemic lupus, rheumatoid arthritis, and inflammatory bowel disease [42–45]. 

The TNF-*α* acts by binding to TNF cellular receptor (TNFR), present on all cells in the body. The family of TNFRs has many members: the first two discovered were the TNFR1 and TNFR2. TNFR2 has a higher affinity for TNF-*α*, especially at lower molecule concentrations, and causes the proliferation of T-lymphocytes and other proinflammatory responses. TNFR1 requires high TNF-*α* concentrations and cause cell death by cytotoxicity and apoptosis [46]. Both the TNFRs are released also in a soluble form that neutralises the TNF-*α* action competing with the cell-bound receptors. At the same time, TNF receptor soluble forms stabilize the TNF-*α* molecules and prevent its degradation. Mutations in the TNFRs are probably involved in autoinflammatory syndromes [47]. 

Generally, higher TNF-*α* levels are related to the severity of inflammatory response, although it is not clear if greater TNF-*α* production causes more severe inflammation or, conversely, if more severe inflammation elicits greater TNF-*α* synthesis. TNF-*α* levels seem to vary too on an individual basis and increase in circulating TNF-*α* levels is much greater in some patients than in others [12].

**Figure 1:**
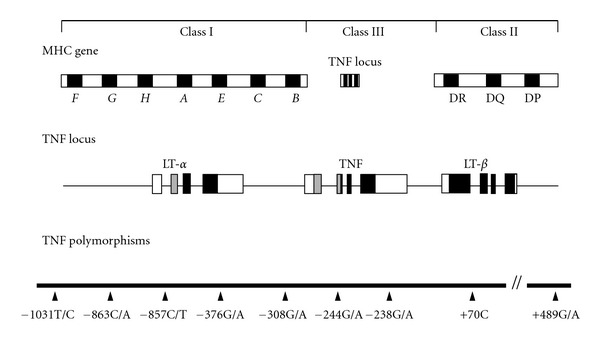
Schematic representation of the location of the TNF gene within the major histocompatibility complex. The position of the most important SNPs in the TNF gene is indicated (adapted from [19]).

The TNF-*α* gene is located on human chromosome 6p21.3 within the major histocompatibility complex (MHC) (Figure 1) [12]. It lies in the so called class I region, between the genes encoding the MHC class human leukocyte antigen (HLA) class II cell surface molecules (HLA-DP, DQ, and DR) and the MHC class I antigen (HLA-A, B, and C). The 5′ flanking region of the TNF-*α* gene contains multiple potential regulatory sites that seems to be responsive to inflammatory stimuli [12, 48].

### 3.3. TNF-*α* Gene Polymorphisms

Genetic factors may affect TNF-*α* levels as showed by *in vitro* and *in vivo* studies. Differences in cytokine production may be partly attributed to the presence of single nucleotide polymorphisms (SNP) within its corresponding gene. At least 12 SNP have been identified in the TNF-*α* locus, some of which have also been shown to influence the rate of transcription and production of TNF-*α* cytokine [49, 50].

The most commonly studied TNF-*α* polymorphism is the −308A/G, also known as TNF 1/2 (rs1800629 [MAF: CEU 0.22 HCB 0.03, JPT 0.02, YRI 0.06]) [6]. The function of this SNP has been suggested by conflicting disease association studies rather than *in vivo/vitro* analysis [51]. The presence of the less common −308A allelic form has been found to be correlated with enhanced spontaneous or stimulated TNF-*α* production [52]. Several studies suggested that the protein preferentially binding to the −308A is likely to be a transcriptional activator, although it has yet to be characterized [50, 53–55]. The less common −308A allele is strongly associated with the MHC haplotype HLA-A1-B8 and DR3, which is in turn associated with high TNF-*α* production and autoimmune disease. This genetic propensity to produce elevated TNF-*α* levels, due to the presence of the −308A polymorphism, may alter the course of an immune response [44, 55, 56]. *In vitro* studies using different techniques (transfection with two variant construct cell lines, allele specific TNF-*α* transcript quantification, −308 tagging SNP within the TNF-*α* primary mRNA transcript) failed to demonstrate function *in vitro* for the −308 TNF-*α* SNP [15, 54, 57]. *In vivo* studies have demonstrated that the −308A TNF-*α* allele had higher transcriptional activity compared with the −308G allele [45, 58]. However, this association has not been found by other authors, probably due to the linkage disequilibrium of truly functional polymorphism with the −308 position and to the variable inclusion of these functional polymorphism in the gene construct [59, 60]. Other differences may be related to the type of cells and of stimuli used in the studies [12].

Another possible functional promoter SNP is the −238G/A (rs361525 [MAF: CEU 0.07 HCB 0.04, JPT 0.00, YRI 0.01]) that is located within the TNF-*α* repressor site, but it has shown contradicting function [61]. Some works demonstrated that the −238A allele is associated with higher TNF-*α* production with respect to the −238G allele [58], but this data is not confirmed by other studies [12, 53, 61–64]. Moreover, Brinkman et al. demonstrated a faster radiological damage in GG patients with respect to the GA genotype [15].

The rare −376G/A (rs1800750 [MAF: CEU 0.01 HCB 0.00, JPT 0.00, YRI 0.01]) is a binding site for the transcriptional factor OCT-1 [65]. OCT-1 seems to remain unbound if the G allele is present. On the other hand, the promoter containing the −376A allele demonstrated a promoter activity superior of 35% compared to the G allele (*P* = 0.002) in a monocyte cell line [12, 66].

Studies regarding the −863C/A (rs1 800630 [MAF: CEU 0.16 HCB 0.18, JPT 0.14, YRI 0.12]) and −857C/T genotypes (rs1199724 [MAF: CEU 0.05 HCB 0.18, JPT 0.11, YRI 0.03]) showed that the rarer A and T alleles provide increased promoter activity and high production of TNF-*α* [6, 32].

Many other promoter variants have been described, as +489, +386, −1301, −857, −419, −376, and −244, but these SNPs are rare, particularly in Caucasian, with conflicting and inconsistent data [6, 19, 32, 53].

### 3.4. TNF-*α* Gene Polymorphisms and Juvenile Arthritis

The involvement of TNF-*α* protein and its receptors in the pathogenesis of JIA has been suggested by many studies [4, 23, 33]. TNF-*α* plays a key function in the initial and prolonged inflammation and in joint destruction, controlling the production of interleukin 1 (IL-1) and other proinflammatory cytokines including interleukin-6 (IL-6) and interleukin-8 (IL-8) [67]. TNF-*α* mediates joint inflammation and destruction by inducing the synthesis and release of inflammatory metalloproteinases, prostaglandins, and nitric oxide in a variety of cell types, as well as inhibiting the production of matrix components [68]. Although there is no evidence of a direct TNF-*α* cytotoxic effect on synovial cells. The role of TNF-*α* in JIA is suggested by the findings of high TNF-*α* levels in the synovial fluids of these patients [69], from studies on transgenic mice overexpressing TNF-*α* and developing a polyarthritis [70], and from the observation of a positive response to anti-TNF-*α* biologic drugs in arthritis patients [71–73]. 

In order to better understand the genetic background of JIA and the role of cytokine SNPs in this disease, several studies have been carried out recently (see Table 1). The role of −308A/G polymorphism in JIA was investigated in many studies. Some authors found that the A allele was significantly more frequent in JIA subjects with respect to controls and was related to a higher disease activity [36] and a poor prognosis [43, 74]. Zeggini et al. showed that the TNF −308A allele is more frequently found in rheumatoid factor positive juvenile polyarthritis and is associated with a more severe disease, while the more common TNF −308G allele may be protective [13]. Modesto et al. [74] found no relationship between genotypes and juvenile arthritis, but −308A was more frequent in systemic JIA subgroup. Other authors found no association between −308A/G genotype and juvenile arthritis [23, 75]. Ozen et al. found that the −308G/A polymorphism was significantly associated with a poor outcome in the Turkish group of JIA patients (*P* = 0.005) but not in the Czech JIA subjects; the authors suggested a possible ethnic allele distribution. Besides, in both JIA cohorts, the distribution of genotypes was not significantly different among different JIA subsets [63]. 

So, most JIA studies are in accord with adult RA results, demonstrating a direct involvement of this polymorphism in the severity of arthritis [42, 76–78].

Some authors demonstrated that the −238G/A allele has a significant association with JIA [75], particularly with persistent oligoarthritis subtype [73]; these data were not confirmed by others [23, 43, 63, 74].

Other SNPs have also been investigated. A study by Date et al. demonstrated that the −863A, −1013C, and −857T alleles were significantly higher in systemic JIA patients with respect to healthy controls. This association was not found in oligoarticular and polyarticular juvenile arthritis subsets. Moreover, the −857T allele seems to enhance the effect of DRB1*0405/DQB1*0401 haplotype in predisposing the development of systemic JIA. Indeed the author suggests that this polymorphism is associated with higher TNF-*α* production [32]. Studies in adult patients have recently demonstrated that −857T allele is an independent risk allele for psoriatic arthritis [79, 80] but similar data were not founded in children. Zeggini et al. examined the association of multiple TNF SNPs (−1031, −863, −857, −376, +489A, +851, +1304) with juvenile oligoarthritis by constructing and analyzing SNP-tagged TNF haplotype in 144 simplex families consisting of parents and affected children. The +489A and the +851A alleles resulted significantly associated with persistent oligoarthritis. No relationship was found for the other SNPs investigated [13, 23, 74]. The +489 polymorphism was found positively associated with radiographic bone damage in studies on adult patients [81]; Oen et al. studied the radiographic joint damage and −308A/G SNP in patients with juvenile arthritis but they didn't find any association, Although the +489 SNP was not investigated in this study [35].

### 3.5. TNF-*α* Polymorphisms and JIA Drug Response

The central role of TNF-*α* in the inflammatory process makes this cytokine an excellent therapeutic target [18]. Germline genetic variability causes variable drug response among individual patients. Knowledge about genetic variants may help to predict drug response or optimal dose in the individual patient [82, 83]. Biologic drugs have been demonstrated effective in the treatment of progressive JIA [18, 84]; however, approximately 20–40% of children affected, especially with the polyarticular and the systemic onset subtypes, have been defined as nonresponders and still have a poor prognosis [37, 85]. As several SNPs have been noted in the TNF-*α* promoter and some reports have shown that production of TNF-*α* is influenced by these SNPs, an association has been suggested between some TNF-*α* promoter SNPs, JIA subtypes, and clinical response to biologic therapy [13, 14, 50].

Etanercept, a fusion protein of extracellular domain of the TNF-*α* receptor combined with the Fc portion of the human immunoglobulin molecule, is the first TNF-*α* antagonist approved for the use in JIA and to date it has proven to be highly efficacious in children with polyarticular JIA [17]. Other important biological agents in JIA are adalimumab, a fully humanized monoclonal antibody, approved for treatment of moderate to severe polyarticular JIA, and infliximab, a chimeric human-murine monoclonal anti-TNF-*α* antibody, not formally approved for JIA patients but commonly used in selected cases [17]. Three studies are actually detectable regarding the influence of TNF-*α* SNPs on the anti TNF-*α* effects in paediatric arthritis population.

To test the influence of TNF-*α* polymorphisms in the etanercept therapy response, Schmeling and Horneff (see Table 2) studied 137 children and founded −308GG genotype in 101, −308AA genotype in 3, and heterozygous in 33 patients. Patients with the −308GG genotype more frequently reached a response to etanercept therapy than patients leading the A allele; the response was most pronounced and significant in patients with rheumatoid factor negative polyarthritis [86]. In contrast, a recent study by Cimaz et al. considered 107 children with different juvenile arthritis subtypes nonresponders to other first line drugs, treated with etanercept (34 patients), infliximab (71 children), and adalimumab (2 subjects). In these patients the authors were not able to find a link between the two TNF-*α* SNPs considered (−238A/G and −308A/G) and clinical response to anti-TNF-*α* [87]. More recently a Serbian group detected the influence of −308A/G TNF-*α* SNP on the metalloproteinase-9 (MMP-9) levels and on the clinical response to etanercept in 66 polyarticular JIA children. They found that patients with the −308GG genotype achieved a clinical response more significant than those with the −308AA genotype (*P* = 0.035) and that MMP-9 levels in patients with the genotype −308GG were significantly decreased after 1 year of treatment with etanercept [88].

Major information can be obtained from studies performed in adults affected by rheumatoid arthritis (RA). It is known that RA and JIA are two distinct entities whereas they have several common characteristics [23]. Works in RA patients investigating response to several anti-TNF-*α* therapy showed some important evidence (see Table 3). A report analysed whether polymorphisms of several cytokine genes are associated with the responsiveness to etanercept treatment in 123 RA patients. Results indicated that 24 patients (20%) were defined as nonresponders. None of the recorded alleles was significantly associated with responsiveness to treatment [85]. No association between −308A/G SNP and therapy response was found also by Ongaro and colleagues; they found that the 676TT genotypes is related to a better response to anti-TNF-*α* drugs with respect to 676TG [89]. Besides, a certain combination of alleles (−308GG) was associated with good responsiveness to etanercept (*P* > 0.05) [20, 90–92]. Another Korean study showed that 70 RA patients with the T allele of TNF promoter SNP −857 responded better to 12 weeks etanercept therapy than homozygous for the C allele [93]. Similar results were found in patients treated with other anti-TNF-*α* drugs. In adult cohorts, Mugnier et al. [94] tested if the −308G/A TNF-*α* SNP influences the response to infliximab therapy in RA patients. According to these authors, patients with −308GG genotype were better infliximab responders and they concluded that this genetic evaluation can be useful for predicting infliximab therapy response. Balog et al. considered the influence of TNF-*α* gene −308G/A polymorphism on therapeutic efficacy of infliximab in patients with RA and Crohn's disease. Most of nonresponders carried the TNF-*α* A allele [95]. Other studies showed that patients carrying the −308G/G allele responded to infliximab treatment better than −308A/G subjects [91, 96, 97]. These results were not found by others [89, 98–101]. Furthermore, Marotte et al. found no association between the −308 SNP and response to infliximab, but the level of circulating TNF-*α* bioactivity resulted was higher in −308A/A or A/G patients than in G/G subjects [102].

Another study in RA patients considered 152 patients subdivided in 3 groups of treatment: adalimumab plus methotrexate, adalimumab plus other DMARDs, and adalimumab alone. The authors studied 3 TNF-*α* SNPs: −308A/G, −238A/G e, −857C/T. At evaluation after 12 weeks of therapy no association between the three TNF-*α* SNPs and clinical response was noted. However, the GGC haplotype (−308G, −238G e, −857C) in a homozygous form presented significant association with lower clinical response in patients on adalimumab plus methotrexate treatment [103]. 

In the biologic era, several studies reported an elevated rate of malignancy in JIA patient treated with TNF-*α* inhibitors and in 2009 the US Food and Drug Administration (FDA) placed a black box warning for these drugs, as result of the identification of 48 malignancies cases occurring in children exposed to anti TNF-*α* biologic drugs [104]. 

Studies in children exposed to biologic drugs reported an increased risk of lymphoma and other cancers with respect to healthy population [105–107]. Nevertheless the expected rate of lymphoma risk in biological treated children is unknown. Moreover the global incidence of cancer in JIA population is not well defined. 

Indeed, two paediatric studies in JIA patient never treated with biologic drugs reported a 2- to 3-fold increased risk of cancer, and in particular 4-fold increased risk of lymphoproliferative disease [108, 109]. Also the work of Beukelman and colleagues showed that JIA children had an increased rate of incident malignancy compared to children with asthma and attention deficit hyperactivity disorders, but the authors showed that specific therapies as methotrexate and TNF inhibitors did not alter this rate [110]. 

A very recent work of Nordstrom et al. considering a cohort of biologics-naïve patients diagnosed with JIA between 1998 and 2007, matched with a non JIA cohort, found that the JIA incidence rates of cancer were significantly higher in JIA with respect controls (67.0 cases/100,000 person-years for JIA and 23.2 cases/100,000 for non-JIA). However, they found a nearly 3-fold increased risk of cancer in biologics-naïve JIA patients [111]. 

In these studies, it is difficult to evaluate the real risk of cancer because of the potential risk of malignancy associated with underlying illnesses and the use of concomitant immunosuppressants; a clear causal relationship could not be established and the findings suggest an elevated underlying risk of cancer in this disease population independently from biologic therapy. 

Paediatric studies confirmed the more consistent data reported in biologic treated adult patients. Wolfe and Michaud found about 3-fold increased risk for lymphoma in 18.572 RA biologic drug treated patients with respect to controls. However, the authors referred that increased lymphoma rates observed with anti-TNF therapy may reflect channeling bias, whereby patients with the highest risk of lymphoma preferentially receive anti-TNF therapy and consequently the data are insufficient to establish a causal relationship between RA treatments and the development of lymphoma [112]. The same authors in a following work, during 89,710 person-years of followup of RA from 1998 to 2005, did not observe evidence for an increase in the incidence of lymphoma among patients who received anti-TNF therapy [113]. Similar results were reported in other several studies [114, 115].

The Italian LOHREN registry reported data in contrast with the previous studies. This work considered 1114 RA patients treated with anti-TNF agents after failing to respond to traditional DMARDs, over an average observational period of 23.32 months. Comparison with the general population showed that the overall cancer risk was similar, but the risk of lymphoma was about five times higher in the RA patients treated with a biological agent [116]. However, even in this study there is the same bias: Patients treated with biologic drugs are the same subjects with severe disease that did not respond to several previous therapies.

The reported studies seem to indicate that not the biologic drug use but the prolonged inflammatory state itself due to the autoimmune disease that can be responsible for the increased risk of cancer [117, 118]. Indeed a recent sponsored Swedish nationwide cohort study found that RA patient anti-TNF-*α* biologic drugs naïve have a significantly higher risk of malignancy compared with general population [119].

At this regard, several studies showed an association between elevated circulating TNF-*α* levels and cancer development [120, 121] stimulating cell proliferation [122], causing DNA damage [123] and promoting angiogenesis [124]. This data was not confirmed by other studies that reported a direct cytotoxic effect of TNF-*α* on tumoral cell [125], an indirect action on tumor vessels [126] and a synergic action with conventional antineoplastic agents [127, 128].

Moreover, a relationship was found between some TNF-*α* polymorphisms and development of different types of cancer. The +488A and the −857T polymorphisms have been associated to bladder cancer [129]. The +488GA genotype seems to be related to development of renal cell carcinoma and prostate carcinoma [130, 131]. The −238GA SNP have been also correlated to renal carcinoma [130] and the +857T SNP to leukemia and lymphoma [132]. The −308G have been found associated with gastric [133], breast [134], and liver cancer [133–136] and represents a negative prognostic factors in pediatric leukemia [137].

Although some biologics seem to have a high association with certain cancer compared to control, there is no consistency of data. Genetic pathways themselves can be also related to higher tumor development risk. So caution is needed in interpreting the data and more research is needing.

## 4. Discussion

The actual knowledge regarding the role of TNF-*α* gene polymorphisms in the pathogenesis of JIA is still incomplete. Numerous studies have focused on understanding the contribution of TNF-*α* polymorphisms in the RA and JIA pathogenesis. 

The TNF-*α* polymorphisms have shown an association with higher or lower levels of circulating TNF-*α*, aggressive or mild disease and poor or good prognosis related to the response to anti TNF-*α* treatment. 

The most studied TNF-*α* SNP is located at −308 position, where the presence of rare A allele was associated with a major gene expression, high level of TNF-*α* expression, and more aggressive JIA phenotypes [36, 43, 74] such as systemic juvenile arthritis and rheumatoid factor positive juvenile polyarticular arthritis [13, 74]. Several data suggest an association of this polymorphism with systemic manifestations, radiological progression, work disability, and joint surgeries [78]. The presence of 308A allele has been moreover linked to increased susceptibility and severity of a variety of other autoimmune disorders including systemic lupus erythematosus [43, 138], dermatomyositis [139, 140], inflammatory bowel disease [141, 142], and asthma [43].

This −308 SNP was found mostly in Caucasian population and represents an important risk factor for JIA appearance in this population. 

Also the −238A TNF-*α* gene polymorphism has been associated with higher TNF-*α* production and more aggressive JIA phenotypes [59, 74, 75]. Moreover these data were not confirmed by others [12, 23, 42, 54, 61–65, 74].

Several other polymorphisms have been identified but their frequency, their pathogenic role, and their influence on biologic drug response have been poorly characterized. 

Results of the studies are mostly conflicting. This is likely related to several elements: the complex pathogenesis of JIA, involving different cytokine genes and non HLA genes, largely still not well defined, and also environmental factors that are, in the major part actually unknown. Anyway the identification of singular patient genetic pattern can change the medical therapeutic approach. TNF-*α* blocking agents are among the most effective therapies for JIA but unfortunately not all patients have a good response. Actually, the reasons for the interindividual variability in the response to anti-TNF-*α* therapy are unclear, although it is supposed that the genetic background might play a role. Considering the increasingly wide range of biologicas available for AIG and the cost of these therapies, there is an increasing need to predict responsiveness to identify patients more suitable to the therapy, to define the timing of treatment, and to avoid complications.

Results of studies in RA patients indicate TNF-*α* as candidate genes potentially involved in the modulation of clinical response to anti TNF-*α* blocking agents. Many studies demonstrated that patients with the −308GG genotype are better responders to anti TNF-*α* therapy. 

Now, conflicting results have been found in the few paediatric studies. Indeed only some authors showed that the −308G/G genotype is associated with a better anti-TNF-*α* treatment response also in JIA [83, 85]. Other authors did not confirm this data [23], therefore adjunctive information are necessary.

To conclude, data gathered so far indicate a possible influence of the −308 SNP promoter position on the production of TNF-*α* and consequently on the severity of JIA and the response to anti-TNF-*α* treatment. Further and larger studies are needed to investigate the influence of TNF-*α* polymorphisms on the treatment response to individualize the management of the disease.

## Figures and Tables

**Table 1:** TNF-*α* SNPs and JIA pathogenesis.

Authors	Date	Population/region	Design	Disease group	Number of subjects	Polymorphisms	Findings
Höhler et al.	1997	Caucasian/Germany	Case control	Juvenile onset Psoriasis (no. 60)		−238	Significant association
				Psoriatic arthritis (no. 62)	122	−308	No association

Date et al.	1999	Japan	Case control	Systemic arthritis (no. 50)		−1031C/T	−1031C, −863A, −857T are significantly higher in case patients particularly in systemic group
				Pauciarthritis (no. 29)	111	−863A/C	
				Polyarthritis (no. 32)		−857T/C	

Ozen et al.	2002	Turkey	Case control	Systemic (no. 9)			−308A is associated with a poor prognosis
				Polyarthritis (no. 20)		−308G/A	
				Persistent oligoarthritis (no. 15)	51		
				Extended oligoarthritis (no. 7)		−238G/A	No association
		Czech Republic	Case control	Systemic (no. 13)			
				Polyarthritis (no. 41)			
				Persistent oligoarthritis (no. 27)	159	−308G/A, −238G/A	No association
				Extended oligoarthritis (no. 19)			
				Enthesitis-arthritis (no. 59)			

						−308G/A	−308A is significantly associated with juvenile oligoarthritis
Zeggini et al.	2004	Caucasian/UK	Case control	Persistent oligoarthritis (no. 92) Extended oligoarthritis (no. 52)	144 simplex families	−238G/A +489G/A +851G/A	−238G, +489A, +851A are significantly associated with *persistent* oligoarthritis
						−1031, − 863, − 857, − 376, + 1304	No association

Miterski et al.	2004	Caucasian/Germany	Case control	JIA	Up to 200	−238A/G, −308A/G, −857C/T	No association

Modesto et al.	2005	Caucasian/Spain	Case control	Oligoarthritis (no. 29) Systemic arthritis (no. 26)	55	308, −238, −376, −163G/A	No significant association, but −308A is more frequent in systemic group

Jimenez-Morales et al.	2009	Mexico	Case control	JIA	171	−308G/A −238G/A	−308A is significantly associated with JIA No association
				Systemic (no. 35)			The T allele demonstrated a significant protective effect against JIA in all groups
				Polyarthritis (no. 131)			
				Oligoarthritis (no. 198)	433	TNFAIP3/rs10499194	
				Enthesitis-arthritis (no. 37)			
				Other (no. 32)			
Prahalad et al.	2009	Caucasian/Utah	Case control	Systemic (no. 36)			The A allele conferred higher risk for JIA
				Polyarthritis (no. 133)			
				Oligoarthritis (no. 199)	441	TNFAIP3/rs6920220	
				Enthesitis-arthritis (no. 40)			
				Other (no. 33)			

Mourao et al.	2009	Portugal	Case control	Systemic (no. 8)			The −308GA/AA allele is related to higher disease activity
				Polyarthritis (no. 27)			
				Oligoarthritis (no. 65)	114	−308G/A	
				Enthesitis-arthritis (no. 9)			
				Psoriatic arthritis (no. 5)			

**Table 2:** TNF-*α* SNPs and JIA therapeutic response.

Authors	Population	Drug (dosage)	Evaluation time	Number of subjects	JIA subtype	Polymorphisms	Findings
					Systemic arthritis (no. 19) FR− polyarthritis (no. 43) FR+ polyarthritis (no. 12) Persistent oligoarthritis (no. 5) Extended oligoarthritis (no. 24) Enthesitis-arthritis (no. 15) Psoriatic arthritis (no. 8) Non classified form (no. 11)	−163, −244, −376	Not found
Schmeling et al. (2006)	Caucasia /Germany	Etanercept	3 months, 6 months, every 6 months thereafter (max 60 months)	137		−238GG versus −238GA/AA	No correlation
						−308GG versus −308GA/AA	−308GG genotype more frequently respond to etanercept therapy, especially in the FR− polyarthritis subgroup until 6 months of therapy.

Cimaz et al. (2007)	Caucasian/ Italy	(i) Infliximab (3 mg/kg) (ii) Etanercept (0.4 mg/kg) (iii) Adalimumab (24 mg/m²)	3 months	107	Systemic arthritis (no. 29) FR− polyarthritis (no. 24) FR+ polyarthritis (no. 5) Persistent oligoarthritis (no. 4) Extended oligoarthritis (no. 27) Enthesitis-arthritis (no. 12) Psoriatic arthritis (no. 6)	−238GG/GA/AA −308GG/GA/AA	No association between SNPs and clinical response to drugs

Basic et al. (2010)	Caucasian/ Serbia	Etanercept	1 year	66		−308G/A	−308GG SNP have response to drug significantly more frequently than −308AA SNP in polyarticular JIA

**Table 3:** TNF-*α* SNPs and RA anti-TNF*α* therapeutic response.

Authors	Number of subjects	Evaluation times	Drug	SNPs	Findings
Padiukov et al. (2003)	123	3 months	Etanercept	−308A/G	Nonsignificant association between genotypes and response to treatment

Mugnier et al. (2003)	59	22 weeks	Infliximab	−308A/G	Patients with −308G/G genotype are better infliximab responders than patients with −308A/A or A/G genotype

Khang et al. (2005)	70	12 weeks	Etanercept	−857C/T	−857T allele is related to a significant better response to etanercept respect to the homozygotes CC allele

Fonseca et al. (2005)	22	56 weeks	Infliximab	−308A/G	After 24.8 weeks of therapy the −308G/G patients had significantly better response than −308A/G subjects

Seitz et al. (2006)	86 (54 RA, 10 psoriatic arthritis, 22 ankylosing spondylitis)	24 weeks	Infliximab (no. 63) Etanercept (no. 13) Adalimumab (no. 10)	−308A/G	Patients with −308G/G genotype are better responders than those with A/A and A/G genotype independent of the treated rheumatic disease

Guis et al. (2007)	86	6 months 12 months	Etanercept	−308A/G	−308G/G genotype is associated with a better response to etanercept respect to −308A/G genotype

Chatzikyriakidou et al. (2007)	58	Retrospective study	Infliximab	−857C/T −308G/A −238G/A 489G>A	No independent polymorphism predict patients' response to anti-TNF-*α* therapy

Micheli-Richard et al. (2008)	380	12 months	(i) Adalimumab + methotrexate (no. 182) (ii) Adalimumab + other modifier drug (no. 96) (iii) Adalimumab (no. 102)	−238A/G −308A/G −857C/T	The −238G/G, −308G/G, −857C/C alleles are significantly associated with a lower response to treatment with ADA + MTX

Pinto et al. (2008)	113	30 weeks		−308G/A −238G/A	No association between genotypes and clinical response to therapy
			Infliximab + MTX		

Marotte et al. (2008)	198	6 months	Infliximab + MTX	−308A/G	The −308 SNP was not associated with the response to infliximab. The level of circulating TNF-*α* bioactivity is higher in −308A/A or A/G patients than that in G/G

Maxwell et al. (2008)	1050	6 months	Etanercept (no. 455) Infliximab (no. 450)	−308A/G −238A/G	The −308AA genotype is significantly associated with a poorer response to ETA with respect to −308GG. This result is not present for Infliximab. The −238GA genotype is associated with a poorer response to Infliximab but not ETA

Ongaro et al. (2008)	105	1 year	Etanercept (no. 55) Infliximab (no. 40) Adalimumab (no. 10)	−676G/T −308A/G	No association was found between −308 genotype and clinical response. The −676TG genotype is significantly associated with a lower response to anti-TNF therapy
